# *RABBIT EARS* regulates the transcription of *TCP4* during petal development in Arabidopsis

**DOI:** 10.1093/jxb/erw419

**Published:** 2016-11-12

**Authors:** Jing Li, Yanzhi Wang, Yongxia Zhang, Weiyao Wang, Vivian F. Irish, Tengbo Huang

**Affiliations:** ^1^Guangdong Provincial Key Laboratory for Plant Epigenetics, College of Life Sciences and Oceanography, Shenzhen University, Shenzhen, Guangdong 518060, P.R. China; ^2^Department of Molecular, Cellular and Developmental Biology, Yale University, New Haven, CT 06520, USA; ^3^Department of Ecology and Evolutionary Biology, Yale University, New Haven, CT 06520, USA

**Keywords:** Arabidopsis, organ growth, petal development, *RABBIT EARS*, *TCP4*, transcription.

## Abstract

Plant organ growth requires the proper transition from cell proliferation to cell expansion and differentiation. The *CIN-TCP* transcription factor gene *TCP4* and its post-transcriptional regulator microRNA319 play a pivotal role in this process. In this study, we identified a pathway in which the product of the C2H2 zinc finger gene *RABBIT EARS* (*RBE*) regulates the transcription of *TCP4* during Arabidopsis (*Arabidopsis thaliana*) petal development. RBE directly represses *TCP4* during the early stages of petal development; this contributes to the role of *RBE* in controlling the growth of petal primordia. We also found that the *rbe-1* mutant strongly enhanced the petal phenotypes of *tcp4soj6* and *mir319a*, two mutants with compromised miR319 regulation of *TCP4*. Our results show that transcriptional and post-transcriptional regulation function together to pattern the spatial and temporal expression of *TCP4*. This in turn controls petal size and shape in Arabidopsis.

## Highlight

*RBE* functions with microRNA319 to control the growth of petals by regulating the transcription of *TCP4* in Arabidopsis.

## Introduction

Plant lateral organs, such as leaves and flowers, initiate on the flanks of the shoot apical meristem as a peg-like primordium (Ha *et al.*, 2010). At early developmental stages, the organ primordium consists of cells that undergo active cell division, which marks the period of ‘growth by cell proliferation’. As the organ grows, cells gradually cease division, generally in a basipetal fashion and enter a phase of post-mitotic expansion, which is described as ‘growth by cell expansion’. In addition, during growth by cell expansion, cells differentiate to form specific structures to allow the organ to carry out its biological functions (Breuninger and Lenhard, 2012; Powell and Lenhard, 2012).

The transition from the period of cell proliferation to expansion and differentiation is precisely controlled by spatial and temporal specific molecular mechanisms, which largely determine the final form of an organ. A number of genes and pathways have been characterized in the regulation of this critical step during organ growth (Powell and Lenhard, 2012). Among these regulators, a suite of genes from the *TEOSINTE BRANCHED1/CYCLOIDEA/PCF* (*TCP*) gene family play a key role (Efroni *et al.*, 2008; Martin-Trillo and Cubas, 2010; Powell and Lenhard, 2012). *TCP* family members encode products that contain a basic helix–loop–helix (bHLH) TCP domain that functions in DNA-binding and protein dimerization. The TCP family is categorized into two classes according to differences within this domain (Kosugi and Ohashi, 2002; Martin-Trillo and Cubas, 2010). Class I *TCP* genes have been proposed to mainly function to promote cell proliferation and concomitant organ growth, while in contrast, Class II *TCP* genes often act as repressors of plant organ growth (Nath *et al.*, 2003; Li *et al.*, 2005; Efroni *et al.*, 2013; Daviere *et al.*, 2014; Schommer *et al.*, 2014).

The *Arabidopsis thaliana* genome contains twenty-four *TCP* genes (Martin-Trillo and Cubas, 2010). Five of them, *TCP2*, *TCP3*, *TCP4*, *TCP10,* and *TCP24* are post-transcriptionally regulated by microRNA319 (Palatnik *et al.*, 2003; Nag *et al.*, 2009). These genes are closely related members in the CIN clade of the Class II TCP family and function together to control a variety of processes in plant development (Koyama *et al.*, 2007; Koyama *et al.*, 2010; Koyama *et al.*, 2011). Among these miR319-regulated genes, *TCP4* has been extensively studied: ectopic expression of a miR319-insensitive *TCP4* (*mTCP4*) gene during early stages of leaf development resulted in the formation of miniature leaves, presumably due to the early onset of a maturation program that precociously terminates cell division in developing leaf primordia (Efroni *et al.*, 2008). Similar phenotypes were also observed in floral organs when *mTCP4* was expressed using a flower-specific promoter (Nag *et al.*, 2009). These phenotypes suggest that *TCP4* represses cell proliferation and promotes post-mitotic differentiation during organ development. Furthermore, it has been suggested that *TCP4* might carry out this role in part by directly activating repressors of cell proliferation, such as the cell cycle inhibitor *ICK1/KRP1* and the miRNA gene *MIR396b* (Rodriguez *et al.*, 2010; Schommer *et al.*, 2014). It has also been shown that *TCP4* regulates the action of the plant hormones auxin, cytokinin and jasmonate, which are implicated in plant growth (Schommer *et al.*, 2008; Efroni *et al.*, 2013).

Apart from miR319 regulation, comparatively little is known of how *TCP4* expression itself is regulated. It has been proposed that the relative levels and domains of expression of *TCP4* and its regulator miR319 are critical for defining *TCP4* activity (Palatnik *et al.*, 2003; Palatnik *et al.*, 2007), indicating that transcriptional and post-transcriptional regulation are both important in controlling the function of *TCP4*. It has also been suggested that the product of the C2H2 zinc finger gene *JAGGED* (*JAG*) might modulate *TCP4* expression but a detailed analysis of this interaction is lacking (Schiessl *et al.*, 2014). In this study, we show that *RABBIT EARS* (*RBE*), which encodes a C2H2 zinc finger transcription factor closely related to *JAG*, directly regulates the transcription of *TCP4* during early petal development in Arabidopsis. *RBE* is specifically expressed in petal primordia at early floral stages. It directly represses the expression of the miRNA gene *MIR164c* that controls the organ boundary regulators *CUP SHAPED COTYLEDON 1* (*CUC1*) and *CUP SHAPED COTYLEDON 2* (*CUC2*) to effect the establishment of petal primordia (Huang *et al.*, 2012). *RBE* also promotes petal primordium growth by directly and negatively regulating the *TCP5* growth repressor gene, which also belongs to the CIN clade of the Class II *TCP* family (Huang and Irish, 2015). *TCP5* has a similar function to *TCP4* in repressing cell proliferation, but is not a target of miR319. During early petal development, *RBE* inhibits the expression of *TCP5* to promote cell proliferation and petal growth (Huang and Irish, 2015). In this study, we show that *RBE* directly associates with the promoter of *TCP4* and acts in concert with miR319 to control *TCP4* expression during early petal development.

## Materials and methods

### Plant materials and growth conditions

*Arabidopsis thaliana* plants were grown under long day conditions (16-hour day/8-hour night) at 22 °C. The *rbe-1* (Takeda *et al.*, 2004) and *mir319a* ^*129*^ (Nag *et al.*, 2009; abbreviated as *mir319a* hereafter) mutants are in the Landsberg *erecta* (L *er*) background. *tcp4soj6* (Palatnik *et al.*, 2007; Nag *et al.*, 2009) and *tcp4-1* mutants (Koyama *et al.*, 2010) were originally in the Columbia (Col) background and were backcrossed with L *er* four times. Homozygous *tcp4soj6* and *tcp4* mutants were identified by genotyping the progeny of the fourth backcross and crossing with *rbe-1* to generate *rbe-1 tcp4soj6/+*, *rbe-1 tcp4soj6* and *rbe-1 tcp4*. The *rbe-1 mir319a* double mutant was made by conventional breeding of both parental lines and confirmed with PCR. Both *mir319a* and *tcp4soj6* seeds were gifts from Dr. Thomas Jack (Dartmouth College, Hanover, NH, USA). *tcp4-1* (GK_363H08) was obtained from the Arabidopsis Biology Resource Center (ABRC). Primers used in genotyping all the mutants are listed in Supplementary Table S1 at *JXB* online.

*35S:GR-RBE* transgenic plants were described previously (Huang *et al.*, 2012). *TCP4p:uidA* is an enhancer trap line (ET5977) in the L *er* background (Sarvepalli and Nath, 2011). This transgenic line was kindly provided by the Cold Spring Harbor Laboratory (http://genetrap.cshl.edu/). *TCP4p:uidA* was introduced into *rbe-1* by crossing to generate *rbe-1 TCP4p:uidA*.

### Dexamethasone (DEX) induction, total RNA extraction and qRT-PCR

In order to induce the function of GR-RBE, DEX (10 μM dexamethasone, 0.1% ethanol, 0.015% silwet) or mock (0.1% ethanol, 0.015% silwet) solution was applied to *35S:GR-RBE* young floral buds. After a 4 hour treatment, floral tissues were harvested and snap-frozen with liquid nitrogen. RNA was extracted with Trizol (Life Technologies), purified using TURBO DNA-free Kit (Life Technologies), and reverse transcribed with Multiscribe reverse transcriptase (Life Technologies) following the manufacturer’s protocols. qRT-PCR was carried out using the Taqman gene expression assay (Life Technologies). Gene expression levels were calculated from three biological replicates using the 2^–ΔΔC^_T_ method (Livak and Schmittgen, 2001). The relative RNA levels were normalized to the value of *ACTIN 2* (*ACT2*).

### Chromatin immunoprecipitation (ChIP)

Chromatin immunoprecipitation (ChIP) was performed according to the previously described protocol (Huang *et al.*, 2012). The *35S:GR-RBE* floral tissues treated with 4 hour DEX or mock were harvested and crosslinked with 1% formaldehyde. Extraction and sonication of nuclei were conducted as in (Huang *et al.*, 2012). In the next step, 10 µL of anti-GR P-20 antibody (Santa Cruz Biotechnology, Dallas, TX, USA) was added to each sample to immunoprecipitate GR-RBE protein. Following reverse-crosslinking and DNA purification, semi-quantitative PCR and qPCR were performed to quantitate the ChIP result. Three regions of the promoter of *TCP4* were examined and the *APETALA3* (*AP3*) exon was used as a negative control. Primers used for PCR are listed in Supplementary Table S1. Semi-quantitative PCR conditions were: 33 cycles, 94 °C for 30 seconds, 55 °C for 30 seconds and 72 °C for 30 seconds. qPCR was carried out using SYBR Green PCR Master Mix (Life Technologies). The fold enrichment of a specific promoter region was determined by first calculating the abundance ratio in DEX- versus mock-treated ChIP samples normalized by the negative control (*AP3* exon) and then divided by the normalized ratio of DEX- to mock-treated input values. Three biological replicates were used for each ChIP experiment.

### Histology and *in situ* hybridization

Detection of β-Glucuronidase activity was conducted as described previously (Nakayama *et al.*, 2005). Whole inflorescences were fixed in 80% acetone at –20 °C for 20 min, then treated with 2 mM X-Gluc in uidA-staining buffer (50 mM sodium phosphate buffer, 10 mM EDTA, 0.1% Triton X-100, 6 mM potassium ferrocyanide and potassium ferricyanide) overnight at 37 °C. After removing the chlorophyll with an ethanol series, inflorescences or individual flowers were mounted in 30% glycerol and examined under a dissecting microscope for whole mount images.

For *in situ* hybridization, the *uidA* coding region was amplified and cloned into pGEM-T Easy vector (Promega, Madison, MI, USA) using primers listed in Supplementary Table S1. The DIG RNA Labeling kit (Roche, Mannheim, Germany) was utilized to synthesize the *uidA* probe. Floral tissues were fixed in freshly made FAA and embedded in Paraplast (Fisher Scientific). 6µm sections were fixed to Probe-on-Plus slides (Fisher Scientific) at 42 °C. *In situ* hybridization and detection all follow the protocols in (Mara and Irish, 2008; Mara *et al.*, 2010).

### Phenotypic analyses

Floral organ number and size were analyzed using the fifth to the twentieth flowers on the inflorescence. Individual floral organs were dissected and photographed for measuring organ width and length in ImageJ. Thirty flowers were counted for floral organ number. Twenty sepals or petals from 10 plants were measured for organ size. Average petal cell sizes were calculated from the number of cells per unit area in ImageJ. Ten petals from 5 plants were used for the cell size measurement. Statistical analyses are described in detail in figure legends.

## Results

### *RBE* regulates the transcription of *TCP4*

**Fig. 1.**
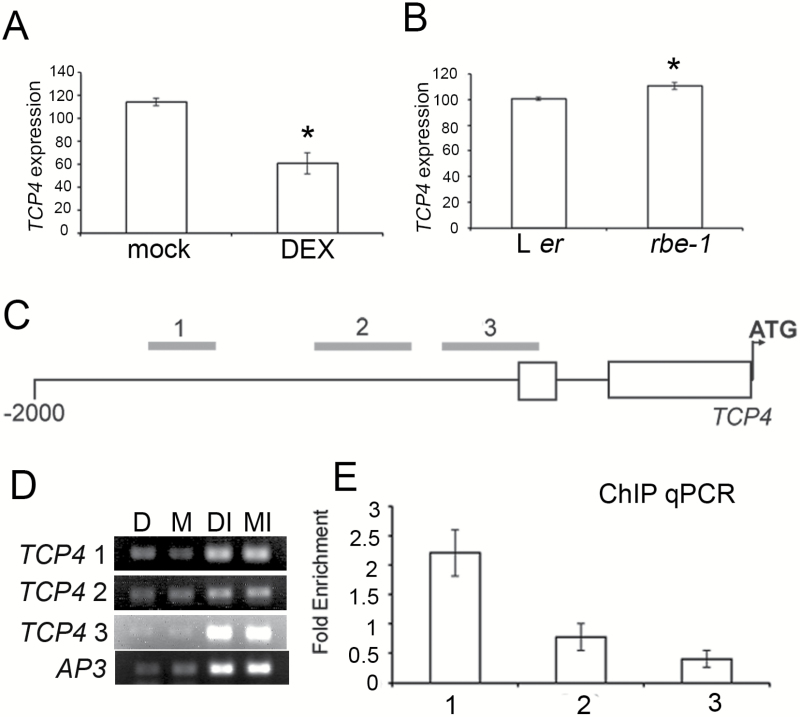
RBE negatively regulates *TCP4* by directly interacting with its promoter. (A) Expression of *TCP4* examined by qRT-PCR in 4 hour DEX- and mock-treated *35S:GR-RBE* flowers. Note that *TCP4* expression is reduced in DEX-treated flowers. (B) Expression of *TCP4* examined by qRT-PCR in L *er* and *rbe-1* flowers. Note that *TCP4* expression is increased in *rbe-1* compared with L *er*. In (A) and (B) the y-axis shows relative expression levels normalized to the value of *ACT2*. Error bars represent stand error of the mean (SEM). Asterisks show a significant difference from the control (*P* < 0.05, Student *t* test). (C) Relative positions of fragment 1, 2, and 3 used for ChIP in the -2000 region upstream of the start codon of *TCP4*. Open boxes indicate 5 prime non-coding exons. (D) Results of semi-quantitative PCR to amplify fragment 1, 2, and 3 in (C) and the control gene (*AP3*). D: DEX-treated samples; M: mock-treated samples. DI and MI indicate input controls. (E) Fold enrichment of fragment 1, 2 and 3 in (C) in DEX- versus mock-treated flowers assessed by ChIP-qPCR after normalizing with input controls. Fragment 1 is approximately 2.5-fold enriched in DEX- versus mock-treatment, consistent with the semi-qPCR result. Error bars represent SEM for three biological replicates.

*RBE* is a key transcriptional regulator of Arabidopsis petal initiation and growth (Takeda *et al.*, 2004; Krizek *et al.*, 2006). We previously performed RNA-sequencing on steroid-inducible *35S:GR-RBE* transgenic plants and characterized components of the downstream gene network responsive to RBE activity (Huang *et al.*, 2012). Among the genes whose expression was significantly reduced upon the induction of *GR-RBE*, we identified *TCP4* as a likely candidate for direct targeting by RBE because of its important function in plant organ growth. We first confirmed the RNA-seq result using qRT-PCR to show that the expression of *TCP4* is decreased in *35S:GR-RBE* floral tissues in the dexamethasone (DEX) treatment as compared to the mock control (Fig. 1A). We also found that the transcript level of *TCP4* is modestly but significantly elevated in the flowers of the *rbe-1* mutant as compared to wild type, which is consistent with a repressive role of RBE on *TCP4* expression (Fig. 1B).

*TCP4* is one of the five *TCP* genes that are post-transcriptionally regulated by microRNA319 (Palatnik *et al.*, 2003; Nag *et al.*, 2009). In order to test whether *RBE* also influences other miR319 target genes, we examined the expression of *TCP2*, *TCP3*, *TCP10*, and *TCP24* in the floral tissues of DEX- and mock-treated *35S:GR-RBE* plants, as well as in *rbe-1* and wild type flowers (see Supplementary Fig. S1). qRT-PCR results showed that DEX-treatment slightly reduced the expression of *TCP3*, *TCP10*, and *TCP24* in *35S:GR-RBE* but had no significant impact on the level of *TCP2* (Supplementary Fig. S1). Furthermore, none of the four *TCP* genes showed a significant change of expression in *rbe-1* as compared to wild type. This indicates that *RBE* does not regulate, or at most plays a minor role, in the regulation of these *TCP* genes (Supplementary Fig. S1). These results all suggested that *TCP4* is a major target of *RBE* among the miR319-regulated *TCP* genes and that *RBE* likely regulates *TCP4* independent of miR319. This is also consistent with our RNA-seq results in that none of the three primary miR319 genes (*MIR319a*/*At4g23713*, *MIR319b*/*At5g41663*, and *MIR319c*/*At2g40805*) were expressed at statistically different levels in *35S:GR-RBE* upon DEX- versus mock-treatment (Huang *et al.*, 2012).

In order to further examine the possible direct association of RBE with the regulatory regions of *TCP4*, we carried out chromatin immunoprecipitation (ChIP) and compared the binding of GR-RBE to the 5′- promoter region of *TCP4* in DEX- and mock-treated conditions. Both semi-quantitative PCR and qPCR revealed a statistically significant enrichment of *TCP4* regulatory sequences in the DEX-treated *35S:GR-RBE* plant extract that was immunoprecipitated with GR antibodies, suggesting that RBE is directly associated with the 5′- regulatory region of *TCP4* and represses the transcription of *TCP4* (Fig. 1D, E).

### *RBE* controls the temporal expression pattern of *TCP4*

**Fig. 2.**
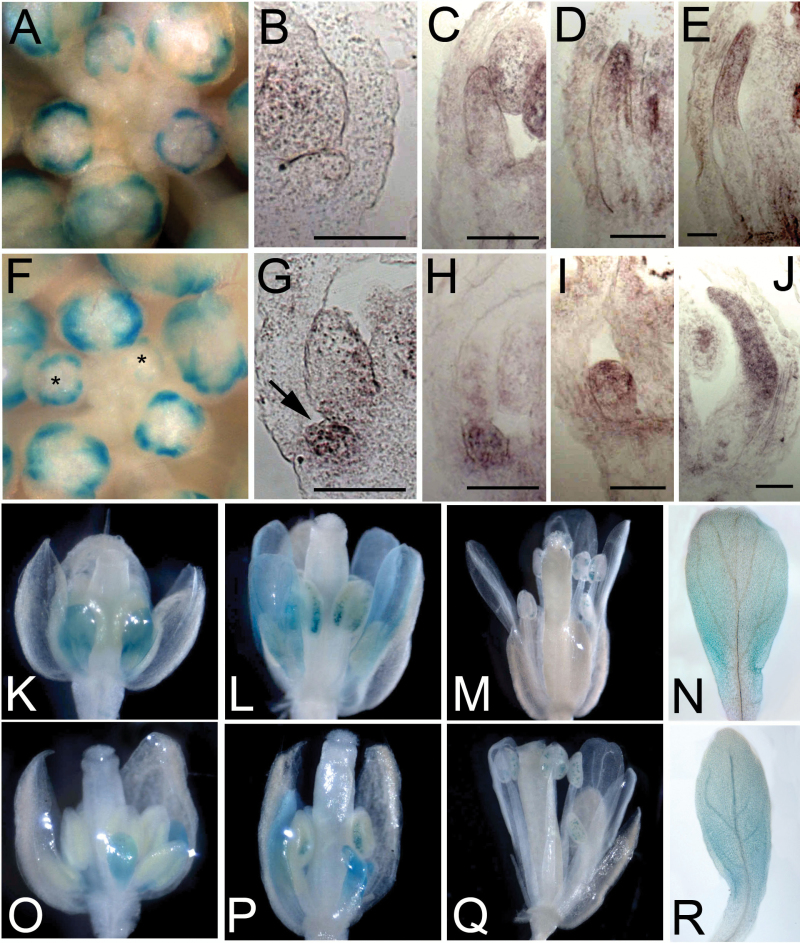
RBE controls *TCP4* promoter activities during petal development. (A) and (F) Whole mount β-Glucoronidase staining shows patterns of *TCP4p:uidA* expressed in the early stage flowers in wild type L *er* (A) and *rbe-1* (F). Note that β-Glucoronidase staining was detected in the presumptive petal primordia at earlier stages (asterisks) in *rbe-1* as compared with wild type. (B) to (E) *in situ* hybridization using the probe that recognizes the *uidA* gene shows the expression of *TCP4p:uidA* in stage 6 (B), 8 (C), 9 (D), 10 (E) petals in L *er*. Note that *TCP4p:uidA* activity starts to be visible in stage 8 petals and is predominantly localized at the distal end of the petal. Bar=50μm. (G) to (J) *in situ* hybridization shows *TCP4p:uidA* expression in stage 6 (G), 8 (H), 9 (I), 10 (J) *rbe-1* petals. Note that *TCP4p:uidA* is precociously expressed in the stage 6 petal (arrow in G) and at a higher level in a stage 8 petal (H) whose growth was strongly reduced. However, *TCP4p:uidA* expression appears to be similar in *rbe-1* and L *er* at later stages of petal development. Bar=50μm. (K) to (M) *TCP4p:uidA* expression shown by whole mount β-Glucoronidase staining in late L *er* flowers at stage 10 (K), 12 (L) and in the open flower (M). (N) Individual petal dissected from (L) shows *TCP4p:uidA* expression at the distal end. (O) to (Q) *TCP4p:uidA* expression shown by whole mount β-Glucoronidase staining in *rbe-1* flowers at stage 10 (O), 12 (P) and in the open flower (Q). Note that *TCP4p:uidA* expression is similar in *rbe-1* and L *er* at these stages. (R) A narrow petal dissected from (P) shows expression of *TCP4p:uidA* at the distal region. (This figure is available in colour at *JXB* online.)

The temporal expression pattern of *TCP4* has been described in detail in leaves and reproductive organs (Palatnik *et al.*, 2003; Sarvepalli and Nath, 2011) but its expression pattern in perianth organs has not been well studied. In order to learn more about the temporal pattern of *TCP4* expression during petal development, we examined the activity of the *TCP4p:uidA* reporter in petal primordia at different floral stages. In wild type L *er* flowers, expression of *TCP4p:uidA* was not detected in the early petal primordia until late stage 8 (Fig. 2A to 2C). From stage 9 to stage 12, *TCP4p:uidA* expression was predominantly localized in the distal region of petals and expression was undetectable in open flowers (Fig. 2A, 2D, 2E, 2K to 2N). In contrast, precocious *TCP4p:uidA* expression in the petal primordia was observed in the *rbe-1* mutant from stage 6 and was relatively stronger when compared to the expression in L *er* prior to stage 9 (Fig. 2F to 2H). However, at later stages of petal development, the expression of *TCP4p:uidA* in the *rbe-1* mutant and L *er* was similar, even in the small and deformed petals (Fig. 2F, 2I, 2J, 2O to 2R). These analyses suggest that *RBE* specifically represses *TCP4* at early stages of petal development.

### *tcp4* partially rescues the mutant phenotypes of *rbe-1* in petal growth

**Fig. 3.**
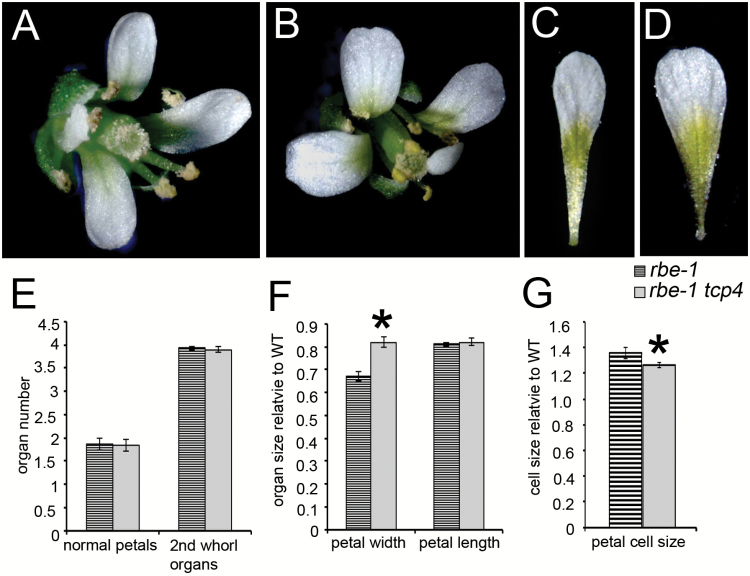
*tcp4* partially rescues the petal phenotypes of *rbe-1*. (A) a *rbe-1* flower, (B) a *rbe-1 tcp4* flower, (C) a *rbe-1* petal, (D) a *rbe-1 tcp4* petal. (E) Numbers of normal petals and second whorl organs in flowers 5–20 for *rbe-1* and *rbe-1 tcp4* (*n*=30; mean±SEM). (F) Measurements of petal width and length in flowers 5–20 for *rbe-1* and *rbe-1 tcp4*. Petal sizes were normalized to the values of the L *er* control (*n*=20; mean±SEM). Asterisks indicate a significant difference between the single and double mutants (*P* < 0.05; one-way ANOVA with Tukey test). See Supplementary Table S2 for the details of statistical analyses. (G) Measurements of petal cell size of *rbe-1* and *rbe-1tcp4*. Cell size was normalized to the wild type value (*n*=10; mean±SEM). Asterisks indicate a significant difference between the single and double mutants (*P* < 0.05, Student *t* test). (This figure is available in colour at *JXB* online.)

To test whether the repression of transcription of *TCP4* is involved in the function of *RBE* in promoting petal growth, we characterized the petal phenotypes of the *rbe-1 tcp4* double mutant. Although the number of ‘normal petals’ - that is petals that have the typical spoon-shape, are white, and are of approximately typical wild type size - and the number of total second whorl organs were similar in *rbe-1* and *rbe-1 tcp4*, the narrow petal phenotype of *rbe-1* was obviously ameliorated in the double mutant (Fig. 3, Supplementary Table S2). Petal width in *rbe-1 tcp4* was significantly increased compared with that of *rbe-1*, which is also consistent with the expression of *TCP4* at the distal region of the petal (Fig. 3C, 3D, 3F, Supplementary Table S2). We also examined cell size in the distal region of petals. *rbe-1* has larger petal cells than wild type (Fig. 3G), which is probably due to compensation for the reduction of cell proliferation in the mutant (Huang and Irish, 2015). In *rbe-1 tcp4*, cell size is slightly but significantly decreased compared with that of *rbe-1*, suggesting that increased cell proliferation is the main reason for the restoration of the petal growth defect in the double mutant (Fig. 3G). These results are consistent with the function of *RBE* and *TCP4* as positive and negative regulators of cell proliferation, respectively (Rodriguez *et al.*, 2010; Schommer *et al.*, 2014; Huang and Irish, 2015) and suggest that the transcriptional repression of *TCP4* plays a role in mediating the function of *RBE* in petal development, especially the lateral growth of the petal blade.

### *rbe-1* enhances the effect of *TCP4* overexpression and miR319 loss-of-function in floral organ development

**Fig. 4.**
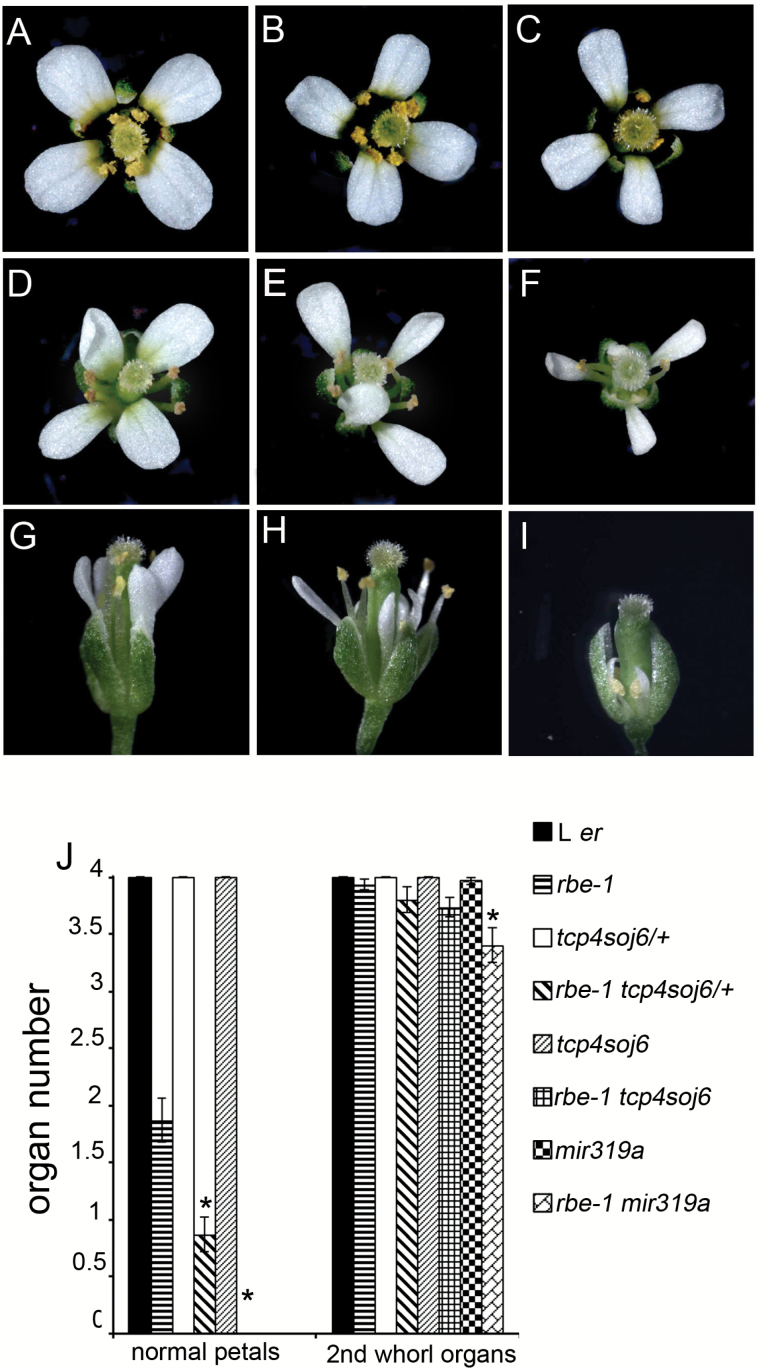
*rbe-1* enhances the petal phenotypes of *tcp4soj6* and *mir319a*. (A) to (C) Flowers of L *er* (A), heterozygous *tcp4soj6* (*tcp4soj6/+*) (B) and homozygous *tcp4soj6* (C). (D) to (F) Flowers of *rbe-1* (D), *rbe-1 tcp4soj6/+* (E) and *rbe-1 tcp4soj6*. (F). (G) to (I) Flowers of *mir319a* (G), *rbe-1 mir319a* with a weaker phenotype (H) and *rbe-1 mir319a* with a strong phenotype (I). All the flowers in (A) to (I) are the fifth flower formed on the inflorescence. (J) Numbers of normal petals and second whorl organs in flowers 5–20 for L *er*, *rbe-1*, *tcp4soj6/+*, *rbe-1 tcp4soj6/+*, *tcp4soj6*, *rbe-1 tcp4soj6*, *mir319a*, and *rbe-1 mir319a* (*n*=30; mean±SEM). Asterisks indicate a significant difference of the double mutant from the corresponding *tcp4soj6/+, tcp4soj6* or *mir319a* single mutant (*P* < 0.05; one-way ANOVA with Tukey test). See Supplementary Table S3 for details of the statistical analyses. (This figure is available in colour at *JXB* online.)

**Fig. 5.**
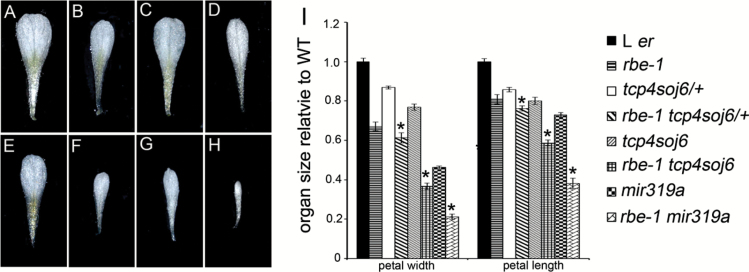
Reduction of petal size in *tcp4soj6* and *mir319a* mutants is largely amplified by the loss of function of *RBE*. Individual petals of L *er* (A), *rbe-1* (B), *tcp4soj6/+*(C), *rbe-1 tcp4soj6/+* (D), *tcp4soj6* (E), *rbe-1 tcp4soj6* (F), *mir319a* (G), and *rbe-1 mir319a* (H). (I) Measurements of petal width and length in flowers 5–20 for L *er*, *rbe-1*,*tcp4soj6/+*, *rbe-1 tcp4soj6/+*, *tcp4soj6*, *rbe-1 tcp4soj6*, *mir319a*, and *rbe-1 mir319a.* Petal sizes were normalized to the values of the L *er* control (*n*=20; mean±SEM). Asterisks indicate a significant difference of the double mutant from the corresponding *tcp4soj6/+, tcp4soj6* or *mir319a* single mutant (*P* < 0.05; one-way ANOVA with Tukey test). See Supplementary Table S4 for details of the statistical analyses. (This figure is available in colour at *JXB* online.)

The function of *TCP4* is controlled by the combined effect of transcriptional and post-transcriptional regulation. This was shown by a partially miR319-resistant form of *TCP4* driven by promoters with different strengths leading to different severities of morphological defects (Palatnik *et al.*, 2007). To test how the ectopic expression of *TCP4* in *rbe-1* influences the effect of *TCP4* in controlling petal growth, we introduced a mutated form of *TCP4* into *rbe-1*. The mutant *TCP4*, *tcp4soj6*, contained a single base pair change that partially disrupts cleavage by miR319 (Palatnik *et al.*, 2007; Nag *et al.*, 2009). Plants heterozygous or homozygous for *tcp4soj6* produced flowers that were morphologically similar to wild type [Fig. 4A to 4C, Supplementary Table S3, (Palatnik *et al*., 2007; Nag *et al*., 2009)]. We only observed a slight reduction in petal size in these mutants (Fig. 4A to 4C, Fig. 5A, 5C, 5E, 5I, Supplementary Table S4 at *JXB* online). However, when combined with *rbe-1*, the double mutants displayed much stronger defects in petal development: both *rbe-1 tcp4soj6/+* and *rbe-1 tcp4soj6* had a significantly decreased number of normal petals compared with their parental lines (Fig. 4B to 4F, 4J, Supplementary Table S3). The measurements of petal size also showed that petal width and length were both reduced in these double mutants (Fig. 5B to 5F, 5I, Supplementary Table S4). In addition, sepal width and length were decreased as well, albeit to a lesser extent, in *rbe-1 tcp4soj6* (Supplementary Fig. S2B to S2F, S2I), supporting a previously suggested non-cell-autonomous function of *TCP4* in growth repression (Nag *et al.*, 2009). Furthermore, petal number and size, and sepal size were all more dramatically affected in *rbe-1 tcp4soj6* compared with *rbe-1 tcp4soj6/+* (Fig. 4E, 4F, 4J, Fig 5D, 5F, 5I, Supplementary Fig. S2D, S2F, S2I, Supplementary Table S3, Supplementary Table S4). This showed that the expression level of *TCP4* is critical for its function in regulating organ growth.

Compared with the weak floral phenotypes of *tcp4soj6* mutants, plants mutant for *MIR319a* showed more severe morphological changes in the flower, particularly in the petals [Fig. 4G, 4J, Fig. 5G, 5I, Supplementary Fig. S2G, S2I, Supplementary Table S3, Supplementary Table S4, (Nag *et al.*, 2009)]. In the double mutant *rbe-1 mir319a*, the floral defects were even more dramatic. Not only did all the petals become small and skinny, the petal loss phenotype was also obvious (Fig. 4G to 4J, Fig. 5G to 5I, Supplementary Table S3, Supplementary Table S4). Petal size was drastically decreased compared with *miR319a*; petals and sepals also became smaller than those from both parental genotypes (Fig. 4G to 4J, Fig. 5G to 5I, Supplementary Table S3, Supplementary Table S4). In some flowers with an extreme phenotype, all the floral organs became small and stunted (Fig. 4I). These results all suggested a strong enhancement of *mir319a* mutant phenotypes by *rbe-1*, which is likely attributable to the alleviation of both transcriptional and post-transcriptional repression of *TCP4* in flowers.

## Discussion

*TCP4* is a major target gene of miR319 in the *CIN-TCP* family. In petal development, overexpression of miR319-resistant m*TCP4* and a mutation in the *miR319a* ^*129*^ allele that reduces the targeting of *TCP4* both resulted in a dramatic reduction of petal growth (Nag *et al.*, 2009). Moreover, the *tcp4soj6* mutant with a mutation in the *TCP4* coding sequence that complements the *miR319a* ^*129*^ allele could largely rescue the *miR319a* ^*129*^ phenotypes, suggesting that *TCP4* is a major downstream effector of *miR319* in petal development (Nag *et al.*, 2009). Among the three genes that encode mature miR319, *MIR319a* was thought to play a more prominent role in the petal. The expression of *MIR319a* partly overlaps with that of *TCP4* in developing petals (Nag *et al.*, 2009), suggesting that miR319 functions to dampen rather than eliminate the transcription of *TCP4*, thus fine-tuning the function of *TCP4* during petal growth.

In this study, we identified another upstream regulator of *TCP4*, *RBE*, which acts in concert with miR319 to control the expression of *TCP4* in petal development. *RBE* encodes a C2H2 zinc finger transcriptional repressor, so it presumably functions via down-regulating the transcription of *TCP4*; this was confirmed by our results that *TCP4* was more highly expressed in the flowers of *rbe-1* compared with wild type (Fig 1). The increase of *TCP4* expression in *rbe-1* is modest, probably because it only occurs at early developmental stages (Fig. 2). However, this specific ectopic expression of *TCP4* might in part be responsible for the petal growth defects in *rbe-1*, as *tcp4* partially rescues the mutant phenotype of *rbe-1* (Fig. 3). This is also consistent with the observation in leaves that excess expression of *TCP4* at early developmental stages results in the reduction of organ growth in the plant (Efroni *et al.*, 2008). Furthermore, we also found that *rbe-1* dramatically enhanced the phenotypes of *tcp4soj6* and *miR319a* ^*129*^, mutants in which the post-transcriptional regulation of *TCP4* was compromised. The strong defects in *rbe-1 tcp4soj6* and *rbe-1 miR319a* further confirmed previous studies showing that transcriptional and post-transcriptional regulation function together to control the level of *TCP4* (Palatnik *et al.*, 2007). A similar means of regulation was also proposed as a key mechanism determining the expression of other miRNA-targeting genes, including *HD-ZIP III* and *CUC* transcription factors (Bao *et al.*, 2004; Sieber *et al.*, 2007).

Despite the prominent effects of miRNA on its targets, identification of the upstream transcriptional regulators is also critical in unraveling the spatial and temporal regulation that patterns the expression of these miRNA-targeting genes. To our knowledge, *RBE* is one of the few transcriptional regulators of *TCP4* that has been functionally characterized in detail. We hope our findings will provide novel insights in uncovering the genetic network that controls this key growth regulating gene and its related developmental processes.

## Supplementary Data

Supplementary data are available at *JXB* online.

Fig. S1: *RBE* has minor and indirect effects on other miR319-regulating *TCP* genes.

Fig. S2: Sepal growth is affected in *tcp4soj6* and *mir319a* mutants and further impaired when combined with *rbe-1.*

Table S1: Primers used in this study.

Table S2: Statistical analyses of the petal size of *rbe-1* and *rbe-1 tcp4.*

Table S3: Statistical analyses of the floral organ number of the single and double mutants of *rbe-1, tcp4soj6/+, tcp4soj6* and *mir319a.*

Table S4: Statistical analyses of the floral organ size of the single and double mutants of *rbe-1, tcp4soj6/+, tcp4soj6* and *mir319a.*

Supplementary Data
