# *RABBIT EARS* regulates the transcription of *TCP4* during petal development in Arabidopsis

**DOI:** 10.1093/jxb/erx036

**Published:** 2017-02-16

**Authors:** Jing Li, Yanzhi Wang, Yongxia Zhang, Weiyao Wang, Vivian F. Irish, Tengbo Huang

**Affiliations:** 1 Guangdong Provincial Key Laboratory for Plant Epigenetics, College of Life Sciences and Oceanography, Shenzhen University, Shenzhen, Guangdong 518060, P.R. China; 2 Key Laboratory of Optoelectronic Devices and Systems of Ministry of Education and Guangdong Province, College of Optoelectronic Engineering, Shenzhen University, Shenzhen, Guangdong 518060, P.R. China; 3 Department of Molecular, Cellular and Developmental Biology, Yale University, New Haven, CT 06520, USA; 4 Department of Ecology and Evolutionary Biology, Yale University, New Haven, CT 06520, USA

*Journal of Experimental Botany*, Vol. 67, No. 22 pp. 6473–6480, 2016 doi:10.1093/jxb/erw419

Jing Li’s affiliation to the Key Laboratory of Optoelectronic Devices and Systems of Ministry of Education and Guangdong Province, College of Optoelectronic Engineering, Shenzhen University, Shenzhen, Guangdong 518060, P.R. China, was missing in the original paper. This has been added above as affiliation 2 and has been corrected in the original article online.

