# Supplementary material for: RABBIT EARS regulates the transcription of TCP4 during petal development in Arabidopsis
Source: J Exp Bot. 2016 Nov 12;67(22):6473–80. doi: 10.1093/jxb/erw419 (PMC5181588; doi:10.1093/jxb/erw419)
Supplement: Supplementary Data [file supp_67_22_6473__index.html]

 RABBIT EARS regulates the transcription of TCP4 during petal development in Arabidopsis — RABBIT EARS regulates the transcription of TCP4 during petal development in Arabidopsis — Supplementary Data 

# *RABBIT EARS* regulates the transcription of *TCP4* during petal development in Arabidopsis

## Supplementary Data

Data files

- supplementary\_figures\_S1\_S2\_tables\_S1\_S4.pdf - Supplementary Data
