# Supplementary material for: RABBIT EARS regulates the transcription of TCP4 during petal development in Arabidopsis
Source: J Exp Bot. 2016 Nov 12;67(22):6473–80. doi: 10.1093/jxb/erw419 (PMC5181588; doi:10.1093/jxb/erw419)
Supplement: Supplementary Data [file supp_erw419_supplementary_figures_S1_S2_tables_S1_S4.pdf]

Supplementary Figure Legends:

Figure S1: RBE has minor effects on other miR319-regulated *TCP* genes

qRT-PCR shows the expression of *TCP2*, *TCP3*, *TCP10*, and *TCP24* in mock- and DEX-treated *35S:GR-RBE* flowers, as well as in L *er* and *rbe-1* flowers. The y-axis is the relative expression level normalized to the value of *ACT2*. Error bars represent standard error of the mean. Asterisks indicate a significant difference from the control ($P < 0.05$, Student *t* test). Note that *TCP2* is not significantly reduced in DEX treatment and expression of all four genes are not increased in *rbe-1* compared to L *er*.

Figure S2: Sepal growth is affected in *tcp4soj6* and *mir319a* mutants and further impaired when combined with *rbe-1*. Sepals of L *er*(A), *rbe-1* (B), *tcp4soj6/+* (C), *rbe-1 tcp4soj6/+* (D), *tcp4soj6* (E), *rbe-1 tcp4soj6 (F)*, *mir319a (G)*, *and rbe-1 mir319a(H)* .

(I) Measurements of sepal width and length in flowers 5-20 for L *er*, *rbe-1*, *tcp4soj6/+*, *rbe-1 tcp4soj6/+*, *tcp4soj6*, *rbe-1 tcp4soj6*, *mir319a*, and *rbe-1 mir319a*. Sepal sizes were normalized to the values of the L *er* control ($n = 20$; mean ± SEM). Asterisks indicate a significant difference of the double mutant from the corresponding *tcp4soj6/+, tcp4soj6*, or *mir319a* single mutant ($P < 0.05$; one-way ANOVA with Tukey test). See Supplementary Table3 for details of statistical analyses.

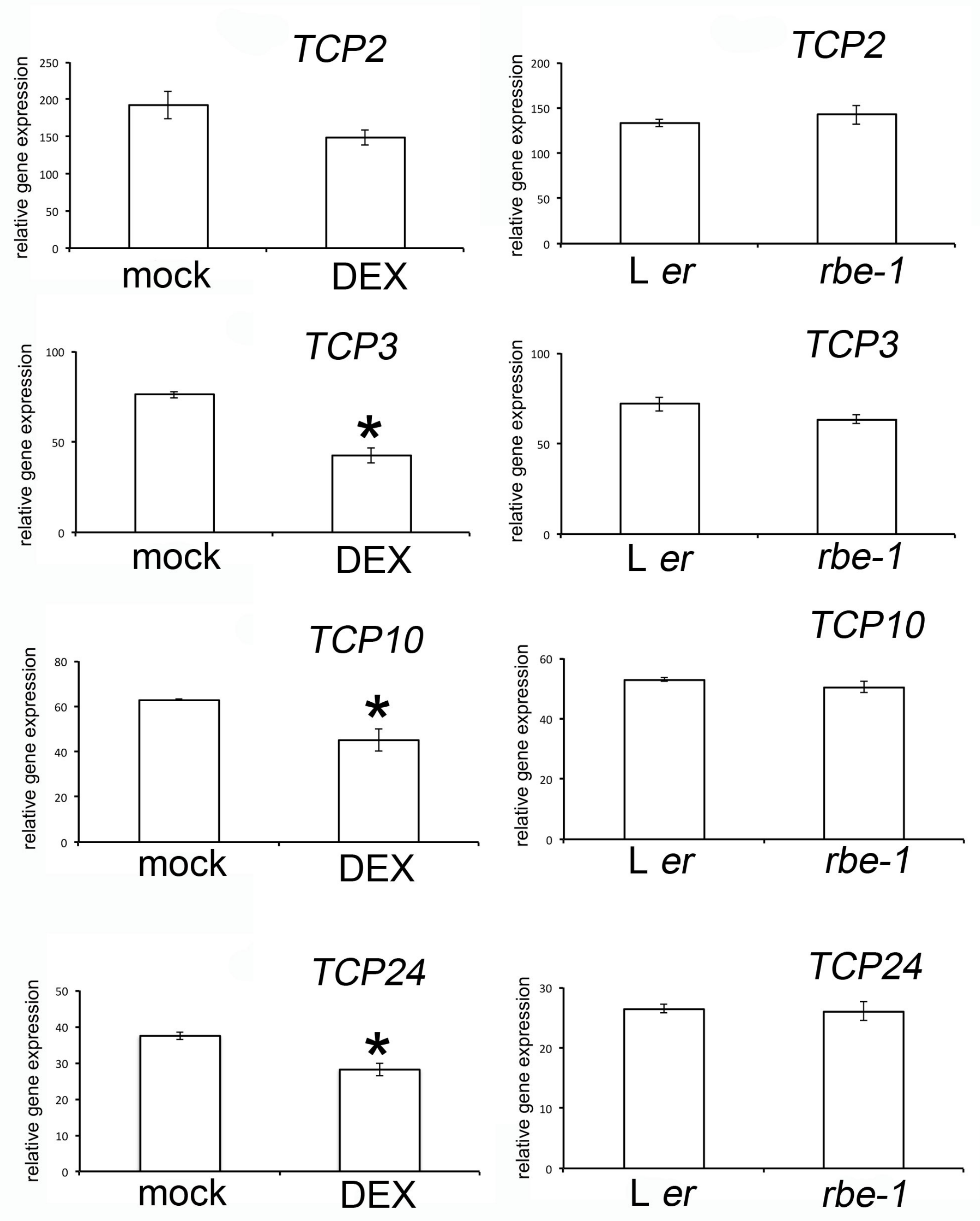

TCP2
relative gene expression
mock
DEX
TCP2
L er
rbe-1
TCP3
*
TCP3
TCP10
*
TCP10
TCP24
*
TCP24

Figure S1

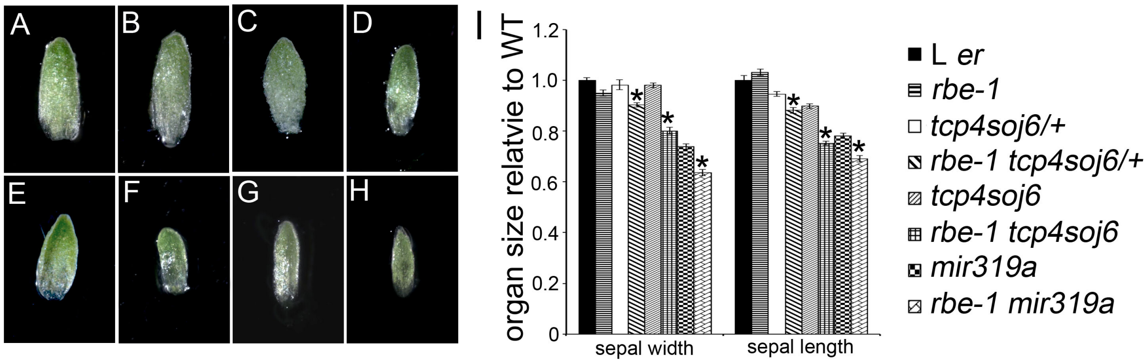
A
B
C
D
E
F
G
H
I
organ size relatvie to WT
1.2
1.0
0.8
0.6
0.4
0.2
0
sepal width
sepal length
L er
rbe-1
tcp4soj6/+
rbe-1 tcp4soj6/+
tcp4soj6
rbe-1 tcp4soj6
mir319a
rbe-1 mir319a

Figure S2

Table S1. Primers used in this study

Primer Name	Primer sequence	Usage	Note
RBE1GF	AGGTCTTACTCATGCAGCTTTTGC	forward primer for genotyping *rbe-1*	PCR product will be digested with DdeI to distinguish WT and *rbe-1*
RBE1GR	AGAACCTTTGATCCCACATCAAGA	reverse primer for genotyping *rbe-1*	
TCP4-1F	ATTCAGTTTTACGATGTTCAAGACAGG	forward primer for genotyping *tcp4-1*	
TCP4-1R	ATCTTGGTTTTGGCTATTAGTTCGAGA	reverse primer for genotyping *tcp4-1*	
TCP4FK	AACAGCTTTATTCTCAGAGGGGTAC	forward primer for genotyping *tcp4soj6*	PCR product will be digested with KpnI to distinguish WT and *tcp4soj6*
TCP4RK	GGCGAGAAATAGAGGAAGCAGAG	reverse primer for genotyping *tcp4soj6*	
MIR319aF	ATTCAATTAGCTTCCGACTCATTC	forward primer for genotyping *mir319a*	PCR product will be digested with BglII to distinguish WT and *mir319a*
MIR319aRB	GAATACAAAAGAGAGAGGGAGATC	reverse primer for genotyping *mir319a*	
qTCP4P1F3	CGGTGAAATCAATTAGTTCCACAAGT	forward primer for ChIP (fragment 1 on the promoter of *TCP4*)	
qTCP4P1R3	TCTTCGTTTTTGATGTTTTTGTTTTT	reverse primer for ChIP (fragment 1 on the promoter of *TCP4*)	
qTCP4P2F	TGCCCATTTTCATATCAAGGAAGT	forward primer for ChIP (fragment 2 on the promoter of *TCP4*)	
qTCP4P2R	TGTGCATGCAAATCATTATGTTATTTT	reverse primer for ChIP (fragment 2 on the promoter of *TCP4*)	
qTCP4P3F	ATTTGGTTGTATTTGCATCTACACATT	forward primer for ChIP (fragment 3 on the promoter of *TCP4*)	
qTCP4P3R	TCGTTTCCCAAATTTATTCATTTTT	reverse primer for ChIP (fragment 3 on the promoter of *TCP4*)	
GUSf	GCCGACAGCAGCAGTT	forward primer for in situ GUS probe	
GUSr	CTCTGGCAACCGGGTGAA	reverse primer for in situ GUS probe	

Table S2. Statistical analyses of the petal size of *rbe-1* and *rbe-1 tcp4*

*: $P<0.05$, one-way ANOVA with Tukey test

n=20	*rbe-1*	*rbe-1 tcp4*
petal width relative to L *er*	0.67	0.82
SEM	0.02	0.02
rbe-1		*
rbe-1 tcp4	*	

n=20	*rbe-1*	*rbe-1 tcp4*
petal length relative to L *er*	0.81	0.82
SEM	0.01	0.02
rbe-1		
rbe-1 tcp4		

Table S3. Statistical analyses of the floral organ number of the single and double mutants of *rbe-1, tcp4soj6/+, tcp4soj6* and *mir319a*

*: *P*<0.05, one-way ANOVA with Tukey test

n=30	L *er*	*rbe-1*	*tcp4soj6/+*	*rbe-1 tcp4soj6/+*	*tcp4soj6*	*rbe-1 tcp4soj6*	*mir319a*	*rbe-1 mir319a*
normal petal number	4.00	1.87	4.00	0.87	4.00	0.00	0.00	0.00
SEM	0.00	0.19	0.00	0.15	0.00	0.00	0.00	0.00
L *er*		*		*		*	*	*
rbe-1	*		*	*	*	*	*	*
tcp4soj6/+		*		*		*	*	*
rbe-1 tcp4soj6/+	*	*	*		*	*	*	*
tcp4soj6		*		*		*	*	*
rbe-1 tcp4soj6	*	*	*	*	*			
mir319a	*	*	*	*	*			*
rbe-1 mir319a	*	*	*	*	*		*	

n=30	L *er*	*rbe-1*	*tcp4soj6/+*	*rbe-1 tcp4soj6/+*	*tcp4soj6*	*rbe-1 tcp4soj6*	*mir319a*	*rbe-1 mir319a*
second whorl organ number	4.00	3.93	4.00	3.80	4.00	3.73	3.97	3.40
SEM	0.00	0.05	0.00	0.11	0.00	0.08	0.03	0.15
L *er*								*
rbe-1								*
tcp4soj6/+								*
rbe-1 tcp4soj6/+								*
tcp4soj6								*
rbe-1 tcp4soj6								*
mir319a								*
rbe-1 mir319a	*	*	*	*	*	*	*	

Table S4. Statistical analyses of the floral organ size of the single and double mutants of *rbe-1, tcp4soj6/+, tcp4soj6* and *mir319a*

*: P<0.05, one-way ANOVA with Tukey test

n=20	L *er*	*rbe-1*	*tcp4soj6/+*	*rbe-1 tcp4soj6/+*	*tcp4soj6*	*rbe-1 tcp4soj6*	*mir319a*	*rbe-1 mir319a*
petal width relative to L *er*	1.00	0.67	0.87	0.61	0.77	0.37	0.46	0.21
SEM	0.02	0.02	0.03	0.02	0.02	0.01	0.03	0.02
L *er*		*	*	*	*	*	*	*
rbe-1	*		*		*	*	*	*
tcp4soj6/+	*	*		*	*	*	*	*
rbe-1 tcp4soj6/+	*		*		*	*	*	*
tcp4soj6	*	*	*	*		*	*	*
rbe-1 tcp4soj6	*	*	*	*	*		*	*
mir319a	*	*	*	*	*	*		*
rbe-1 mir319a	*	*	*	*	*	*	*	

n=20	L *er*	*rbe-1*	*tcp4soj6/+*	*rbe-1 tcp4soj6/+*	*tcp4soj6*	*rbe-1 tcp4soj6*	*mir319a*	*rbe-1 mir319a*
petal length relative to L *er*	1.00	0.81	0.86	0.76	0.80	0.59	0.73	0.38
SEM	0.02	0.01	0.01	0.01	0.02	0.02	0.01	0.03
L *er*		*	*	*	*	*	*	*
rbe-1	*					*	*	*
tcp4soj6/+	*			*		*	*	*
rbe-1 tcp4soj6/+	*		*			*		*
tcp4soj6	*					*	*	*
rbe-1 tcp4soj6	*	*	*	*	*		*	*
mir319a	*	*	*		*	*		*
rbe-1 mir319a	*	*	*	*	*	*	*	

n=20	L *er*	*rbe-1*	*tcp4soj6/+*	*rbe-1 tcp4soj6/+*	*tcp4soj6*	*rbe-1 tcp4soj6*	*mir319a*	*rbe-1 mir319a*
sepal width relative to L *er*	1.00	0.95	0.98	0.90	0.98	0.80	0.74	0.64
SEM	0.01	0.01	0.02	0.01	0.01	0.01	0.01	0.01
L *er*				*		*	*	*
rbe-1						*	*	*
tcp4soj6/+				*		*	*	*
rbe-1 tcp4soj6/+	*		*		*	*	*	*
tcp4soj6				*		*	*	*
rbe-1 tcp4soj6	*	*	*	*	*		*	*
mir319a	*	*	*	*	*	*		*
rbe-1 mir319a	*	*	*	*	*	*	*	

n=20	L *er*	*rbe-1*	*tcp4soj6/+*	*rbe-1 tcp4soj6/+*	*tcp4soj6*	*rbe-1 tcp4soj6*	*mir319a*	*rbe-1 mir319a*
sepal length relative to L *er*	1.00	1.03	0.95	0.88	0.90	0.75	0.78	0.69
SEM	0.02	0.01	0.01	0.01	0.01	0.01	0.01	0.01
L *er*			*	*	*	*	*	*
rbe-1			*	*	*	*	*	*
tcp4soj6/+	*	*		*		*	*	*
rbe-1 tcp4soj6/+	*	*	*			*	*	*
tcp4soj6	*	*				*	*	*
rbe-1 tcp4soj6	*	*	*	*	*			*
mir319a	*	*	*	*	*			*
rbe-1 mir319a	*	*	*	*	*	*	*	
